# Integrity of Sperm Cell Membrane in the Semen of Crossbred and Purebred Boars during Storage at 17 °C: Heterosis Effects

**DOI:** 10.3390/ani11123373

**Published:** 2021-11-25

**Authors:** Anna Wysokińska, Dorota Szablicka

**Affiliations:** Faculty of Agrobioengineering and Animal Husbandry, Siedlce University of Natural Sciences and Humanities, 08110 Siedlce, Poland; dorotaszablicka@o2.pl

**Keywords:** crossbred boars, cell membrane structure, heterosis, semen quality, semen storage time

## Abstract

**Simple Summary:**

The cell membrane of spermatozoa is the main structural element of these gametes. In boars, due to its structure, it is most susceptible to various types of damage induced by various factors. Artificial insemination in pigs mainly involves the use of liquid semen preserved at 17 °C. Thus, it is important to monitor this semen during its storage. In practice, the changes that can take place in sperm during the preservation and storage of boar semen are not analysed. Furthermore, considerable variation is observed in the characteristics of boar semen, which may depend on the breed or crossbreeding variant of the boar. Crossbred boars are often used in artificial insemination, because they not only easily produce ejaculates with good parameters, but also have good libido characteristics. However, despite the benefits of artificial insemination with semen of crossbred boars, there is insufficient knowledge of the sensitivity of cell structures to conditions associated with semen storage in comparison with boars of the parent breeds. For this reason, a study was conducted to analyse changes in the integrity of sperm cell membranes taking place during the storage of semen collected from Duroc × Pietrain crossbred boars and purebred boars of the parent breeds. The sperm of Duroc × Pietrain crossbred boars were found to be less sensitive to the conditions of semen storage and to better retain cell membrane integrity than the sperm of purebred males, which was confirmed by calculating the heterosis effects for semen assessed at different hours of storage at 17 °C.

**Abstract:**

The aim of the study was to assess changes in the integrity of sperm cell membranes during the storage of semen collected from Duroc × Pietrain crossbred boars and purebred boars of the component breeds. To compare the cell membrane integrity of sperm heads in crossbred and purebred boars, heterosis effects were estimated. The study was conducted on 48 ejaculates collected from Duroc × Pietrain crossbred boars and from purebred Duroc and Pietrain boars used for artificial insemination. Microscope slides were prepared from each ejaculate for the evaluation of the cell membrane integrity of the sperm, at 1, 24, 48, 72, and 96 h after collection of the ejaculate. Diluted ejaculates were stored at 17 °C. Sperm membrane integrity was analysed by two methods: SYBR-14/PI and eosin–nigrosin. Our results showed that the cell membrane integrity of sperm heads changed with storage time, but the extent of the changes varied depending on the genetic group of boars. The semen of Duroc × Pietrain crossbreds was clearly seen to be less sensitive to storage conditions than that of boars of the parent breeds, which was confirmed by the calculated heterosis effects. The percentage of sperm with an intact cell membrane was higher in crossbred boars than in purebred boars (*p* ≤ 0.05). In addition, significantly fewer moribund sperm spermatozoa and spermatozoa with a damaged cell membrane were observed in crossbred boars (*p* ≤ 0.05). In the semen of purebred Duroc and Pietrain boars, the cell membrane integrity of the sperm should be assessed more often during storage than in the semen of Duroc × Pietrain crossbred boars. This study provides valuable information for the development and implementation of semen quality monitoring in crossbred boars and boars of the parent breeds during storage at 17 °C with respect to the cell membrane structure of sperm heads. The evaluation methods used effectively identify damage to the cell membranes of the sperm during semen storage.

## 1. Introduction

Spermatozoa are gametes that differ from somatic cells in many ways, such as in their structure. A normal sex cell structure, including that of spermatozoa, is essential for successful fertilization [1]. The cell membrane of the sperm head is a structural element of the sperm cell, which often undergoes deformation due to various factors. Due to its structure, the cell membrane of the boar sperm is more susceptible to various types of damage compared with that of the sperm of other species [2,3]. It contains many polyunsaturated fatty acids [4] and a low level of cholesterol in relation to phospholipids [5]. The integrity of the sperm cell membrane is one of the most important parameters taken into account in the assessment of sperm quality for predicting male fertility [6]. The integrity and normal functioning of the sperm cell membrane are essential for sperm metabolism, capacitation, acrosome reaction, and the ability to fertilize the ovum [7]. Factors such as the rate of cooling [8], the dilution procedure [9], and storage conditions [10,11] can adversely affect the structure of the sperm cell membrane. Cooling induces changes in the sperm cell membrane, impairing its functional and molecular state [2,8,12]. Its susceptibility, however, depends on numerous environmental and genetic factors. Many studies have shown that the morphology and morphometry of the boar sperm are influenced by the animal’s breed [13] and age [14,15]. The differences observed in sperm structure are also determined by the type of breed used for the crossing or individual variation in a given breed [16,17]. Some studies have shown that crossbred boars have better semen characteristics than purebred boars [18], as evidenced by the heterosis effects determined for the physical parameters of ejaculates [18,19]. Crossbred boars are often used in artificial insemination, because they not only easily produce ejaculates with good parameters, but also have good libido characteristics. However, despite the benefits of artificial insemination of crossbred boars, there is insufficient knowledge of the sensitivity of cell structures to conditions associated with semen storage in comparison with boars of the parent breeds. In terms of practical use of semen, a thorough assessment of the individual elements of the sperm cell is important. Artificial insemination in pigs mainly involves the use of liquid semen preserved at 17 °C [20]. Thus, it is important to monitor this semen during its storage. In practice, the changes that can take place in the sperm during the preservation and storage of boar semen are not analysed. Objective methods for evaluating sperm structures involve the use of fluorescent dyes [21,22], which stain the structure and make it possible to determine the degree to which it is normal. These stains are increasingly used to supplement traditional diagnostic methods of semen evaluation.

In the present study, an assessment was performed on changes in the cell membrane integrity of the sperm during the storage of semen collected from Duroc × Pietrain crossbred boars and purebred boars representing the parental components. To compare the cell membrane integrity of the sperm heads in crossbred and purebred boars, the effects of heterosis were determined.

## 2. Materials and Methods

### 2.1. Animals, Semen Collection, and Storage Time

The study was conducted on 48 ejaculates (16 from each breed) collected from 24 fertile boars, represented by 8 purebred Duroc boars, 8 purebred Pietrain boars, and 8 two-breed Duroc × Pietrain crosses remaining in service in an insemination station. The boars were 18–24 months of age. The boars were kept in single pens, had uninterrupted access to water, and were ensured appropriate welfare conditions. Two ejaculates were collected from each boar by manual method at an interval of 5 days. Immediately after collecting the ejaculate, the basic parameters (volume, motility, sperm concentration, and percentage of sperm with normal morphology) were determined. The ejaculates that were included in the study had at least 70% progressive sperm and at least 85% normal sperm morphology. Sperm motility was evaluated with a Nikon Eclipse 50i light microscope equipped with a heated stage. A sample of 5 μL of sperm suspension was placed on a prewarmed slide and sealed with a coverslip at 37 °C. Under 200× magnification, the percentage of normally motile spermatozoa was determined in the overall number of sperm present in the field of vision of the microscope. Sperm concentration in the ejaculates was determined with a photometric method using a spectrophotometer (IMV Technologies, l’Aigle, France). The ejaculates were diluted with a Biosolvens Plus (Biochefa, Sosnowiec, Poland) semen extender. The dilution factor of the ejaculate was determined by the concentration of the sperm. A one-step dilution with a diluent heated to 32 °C was used. The ejaculates were diluted with a Biosolvens Plus (Biochefa, Sosnowiec, Poland) semen extender and then stored as a liquid divided into 90 mL doses with a concentration of 3 × 10^9^ spermatozoa per dose. The insemination doses were stored at 17 °C in the refrigerating chamber. The analyses were conducted at 1, 24, 48, 72, and 96 h of storage. A different insemination dose was opened for each analysis in order to prevent microbial contamination. Two microscope slides were prepared from each dose, and each was stained by a different technique.

### 2.2. Staining Methods

The SYBR-14/PI and eosin–nigrosin staining methods were used.

#### 2.2.1. SYBR-14/PI Staining Method

The specimens were stained using the Live/Dead Sperm Viability Kit (Molecular Probes Inc., Leiden, The Netherlands). A 5 μL volume of 50× diluted SYBR-14 was added to 1 mL of diluted ejaculate, followed by incubation at 36 °C for 10 min. Then 5 μL of propidium iodide (PI) was added, followed by incubation at 36 °C for 10 min. A drop of solution was applied to a heated microscope slide, and the integrity of the cell membranes was examined using a Nikon Eclipse 50i microscope with a fluorescence. One slide per sample were analyzed. On each slide, 200 sperm were evaluated. Sperm emitting green fluorescence over the entire head were identified as live cells (with an intact cell membrane stained by SYBR-14), sperm emitting red fluorescence over the entire head or on part of the head and sperm emitting yellow-orange fluorescence over the entire head were identified as dead (with a damaged cell membrane, stained by PI), and sperm emitting yellow-orange fluorescence over the entire head were identified as moribund sperm.

#### 2.2.2. Eosin–Nigrosin Staining Method

Smears were prepared as follows: a drop of semen (5 µL) was applied to a microscope slide heated to about 40 °C and mixed with a drop of stain (5% eosin B solution (Carl Roth GmbH + Co. KG, Karlsruhe, Germany) and 10% aqueous solution of nigrosine (Sigma-Aldrich, St. Louis, MO, USA) mixed in a 1:4 ratio), twice as large, using a glass rod. One slide per sample were analyzed. On each slide, 200 sperm were evaluated, distinguishing sperm with a normal cell membrane structure that remained unstained (live) and sperm with a damaged cell membrane structure, stained pink (dead).

### 2.3. Statistical Analysis

**Figure 1:**
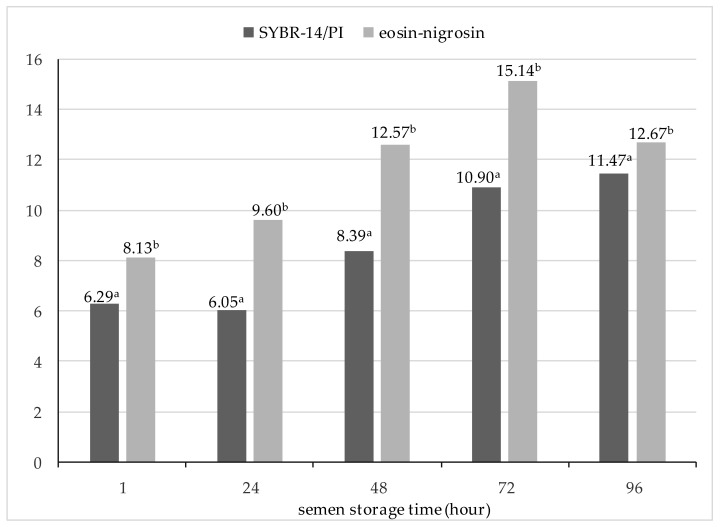
Effects of heterosis calculated for the integrity of the sperm membrane of sperm stained with SYBR-14/PI and eosin–nigrosin. Bars with different letters mean statistically significantly different values (*p* ≤ 0.05).

The results are presented as mean ± standard error of the mean (SEM). Analysis of variance of the results was performed using Statistica v. 13.1 software (StatSoft, Tulsa, OK, USA). Statistical analysis of the material was performed according to the following mathematical model: Y_ij_ = µ + a_i_ + e_ij_ where Y_ij_ is the value of trait, µ is the population mean, a_i_ is the effect of boar breed (Table 1, Table 2, Table 3 and Table 4) or the effect of the staining method (Figure 1), and e_ij_ is error.

The significance of the differences between groups was determined using Tukey’s test at *p* ≤ 0.05.

The heterosis effects for the cell membrane integrity of the sperm of Duroc × Pietrain crossbred boars in relation to the mean for the trait in boars of the parent breeds (Duroc and Pietrain) were calculated according to the following formula:

VR = (X_F1_ − X_MP_)/X_MP_ × 100 (1)

where:

VR is the effect of heterosis;

X_F1_ is the mean for trait in Duroc × Pietrain crossbred boars;

X_MP_ is the mean for trait in boars of the parent breeds (Duroc × Pietrain) [17].

## 3. Results

The percentages of the integrity of the sperm membrane as determined by the SYBR-14/PI method in the semen of Duroc × Pietrain crossbred boars and purebred Duroc and Pietrain boars at different hours of semen storage are presented in Table 1. The data indicate that the semen of crossbred boars had a higher percentage of sperm with an intact cell membrane than the semen of purebred boars (*p* ≤ 0.05). This was observed at every hour of semen storage and is confirmed by the heterosis effects, which ranged from 6.05% at 24 h of storage to 11.47% at 96 h of storage. However, with the storage time, the proportion of sperm with a normal cell membrane structure decreased. The tendency of the integrity of the sperm membrane to decrease with storage time was noted in all breed groups, but in varying degrees. At 96 h of storage, the lowest integrity of the sperm membrane structure was noted in the semen of Duroc boars; it was 10.25% lower than in Pietrain boars and 11.75% lower than in Duroc × Pietrain crossbred boars (*p* ≤ 0.05). The heterosis effects calculated for the integrity of the sperm membrane at 72 and 96 h of storage exceeded 10%.

Table 2 presents the integrity of the sperm membrane and the effects of heterosis in the semen of crossbred boars and purebred Duroc and Pietrain boars depending on the storage time. These results were obtained by evaluating slides stained by the eosin–nigrosin method. The data indicate that the spermatozoa of crossbred boars have better cell membrane integrity than the sperm of purebred boars (*p* ≤ 0.05). The heterosis effects were high, ranging from 8.13% at 1 h of storage to 15.14% at 72 h of storage. The semen of crossbred boars are clearly seen to be less sensitive to semen storage conditions than the semen of purebred boars, as evidenced by the effects of heterosis. From 48 h of preservation of the semen of crossbred boars, the effects of heterosis were relatively large, at over 12%. At 72 h of storage, the frequency of sperm with an intact cell membrane in Duroc × Pietrain boars was 12.75% higher than in Duroc boars and 10.50% higher than in Pietrain boars, and the heterosis effect was 15.14%—its highest value among all storage times.

SYBR-14/PI staining was used to identify sperm with an abnormal cell membrane structure (Table 3) and moribund sperm (Table 4) in the semen of crossbred and purebred boars. The ejaculates of Duroc × Pietrain crossbreds were shown to have a higher percentage of sperm with a damaged cell membrane than the ejaculates of Pietrain boars, but lower than those of Duroc boars (Table 3). However, the averages for sperm with an abnormal cell membrane structure in boars of both parent breeds combined were higher than in crossbred boars, as confirmed by the heterosis effects, which in most cases were negative. Only at 24 h of semen storage was the percentage of sperm with an abnormal cell membrane structure higher in crossbred boars than in boars of the parent breeds (VR = 5.99%).

There were significantly fewer moribund sperm in crossbred boars than in purebred boars (Table 4). The percentage of such sperm was lower in crossbred boars at every hour of semen storage (*p* ≤ 0.05). The percentage of moribund sperm increased with the storage time of the semen of crossbred boars and the semen of Pietrain boars. In boars of the Duroc breed, in the first 48 h of semen storage, there was an increase in the number of moribund sperm, followed by a decrease at subsequent hours of storage.

Figure 1 illustrates the heterosis effects for the percentage of sperm with an intact cell membrane as determined by the SYBR-14/PI and eosin–nigrosin staining methods at different hours of semen storage. The data indicate that the heterosis effects determined in semen stained by the eosin–nigrosin method were higher than in the case of SYBR-14/PI staining (*p* ≤ 0.05). Heterosis effects calculated for the integrity of the sperm membrane determined by the eosin–nigrosin staining method were greatest at 72 h of storage, while in sperm stained by the SYBR-14/PI method, heterosis effects were greatest at 96 h of storage.

## 4. Discussion

The study showed that the integrity of sperm cell membranes changes with the storage time of the semen, but to varying degrees in different breed groups of boars. The semen of Duroc × Pietrain crossbred boars was clearly seen to be less sensitive to storage conditions than the semen of boars of the parent breeds, as confirmed by the heterosis effects. This is a very important observation for the practical use of boars of various breed groups in artificial insemination. Some studies have shown that the ejaculates of crossbred boars have better quantitative and qualitative parameters [23], as indicated by the heterosis effects for these traits [18,24]. Our study showed that the integrity of the sperm membrane decreases with the semen storage time. This tendency was noted in the case of sperm stained with SYBR-14/PI as well as sperm stained with eosin–nigrosin.

The present study showed that in purebred Duroc and Pietrain boars, the cell membrane structure of the sperm head becomes damaged more quickly during semen storage than in crossbred boars. This indicates that the sperm of purebred breeders are more sensitive to the environmental factors to which semen is exposed after it is collected. Many studies have shown that various exogenous factors can affect the quality of boar sperm during the stages of laboratory processing [2,9] and during storage [10,20,25]. Therefore, the sperm of crossbred boars appear to show greater resistance than the sperm of boars of the parent breeds. For this reason, variation in semen quality stemming from the breed of the boar should be taken into account in artificial insemination practice, and sperm structures should be evaluated during preservation, particularly in the case of purebred boars. The ejaculate traits of boars have been shown to vary widely due to the effect of a variety of factors, both genetic and environmental [23,26]. Therefore, the sperm of some males may be more resistant to semen processing techniques, such as extension or storage. This was clearly seen in our study in the case of crossbred boars, and was also confirmed by the heterosis effects calculated for the integrity of the sperm cell membranes.

The ejaculates of Duroc × Pietrain crossbred boars are usually of better quality than those of purebred Duroc and Pietrain boars [27,28], as evidenced by heterosis effects, indicating the advantage of crossbreds over purebred breeders [18,19,29]. The ejaculates of crossbred boars are also observed to have less favourable traits than those of purebred breeders [30], or traits with intermediate values between those of the parent breeds [23]. Therefore, it seems that in order to achieve favourable crossbreeding effects, appropriate breeds must be selected for crossing. The present study was conducted on Duroc × Pietrain crossbred boars, which indicates that the choice of breeds for crossbreeding is highly favourable and enables efficient exploitation of the boars for artificial insemination.

Due to the importance of the evaluation method in the practice of artificial insemination using liquid boar semen, in the present study, we used two methods to evaluate the integrity of cell membranes: a method using fluorochromes (SYBR-14/PI) and the eosin–nigrosin method. Two fluorochromes are used in the SYBR-14/PI method: SYBR-14, which is able to penetrate the intact cell membrane and bind to the nucleic acids of the nucleus of live cells, emitting green fluorescence [26,31], and propidium iodide, which binds to the DNA of dead cells with a damaged cell membrane, emitting red fluorescence [12,32]. Some studies have shown that the percentage of sperm with an intact cell membrane as determined by fluorescence staining is usually lower than in the case of the eosin–nigrosin method [33,34]. In the present study as well, the percentage of sperm with an undamaged cell membrane stained with SYBR-14/PI fluorochromes was lower than in the case of the eosin–nigrosin staining method. The frequency of such sperm, however, changes depending on the semen storage time, as confirmed by both diagnostic methods used to identify sperm with an intact cell membrane. It should be emphasized, however, that the extent of changes in the percentage of sperm with an intact cell membrane observed at different hours of storage differed between crossbred and purebred boars. In the semen of Duroc boars, the decrease in the percentage of sperm with a normal cell membrane structure was greater than in crossbred boars and boars of the Pietrain breed.

The cell membrane is an outer cell structure of the sperm head, which is of fundamental importance for the fusion of the spermatozoon with the oocyte. An intact sperm plasma membrane is a condition for the normal functioning of the cell and for each stage essential to the fertilization of the oocyte [35]. Due to its specific structure in boar sperm, it is more sensitive to all types of procedures performed on the semen of this species. Cholesterol and phospholipids are well known to be basic components of the cell membranes of sperm. Cell membrane integrity can be preserved by maintaining the appropriate physiological ratio of cholesterol to phospholipids [36]. Some studies indicate that the interaction between cholesterol and phospholipids can prevent premature capacitation through the stabilization of sperm cell membranes [37]. Differences have been shown in the cell membrane composition of boar sperm, particularly in the content of unsaturated fatty acids and cholesterol [38]. The cell membranes of boar sperm contain relatively small amounts of cholesterol, and thus are highly sensitive to cold [39]. In addition, prolonged ejaculate processing time has been shown to result in a loss of sperm cell membrane integrity [40]. The cooling process also significantly affects the quality of preserved semen [12]. Sperm cell membranes are highly sensitive to temperature changes during both storage and cooling [41]. In the temperature range of 30 to 10 °C, a change in the lipid phase takes place in sperm cell membranes [8], and the permeability of cell membranes may increase in this temperature range [39]. In consequence, the properties of sperm cell membranes and their capacity to adapt to storage may deteriorate [42].

## 5. Conclusions

To conclude, the sperm of Duroc × Pietrain crossbred boars retain cell membrane integrity to a greater degree than the sperm of purebred males, which was confirmed by calculating the heterosis effects for semen evaluated at different hours of storage at 17 °C. The cell membrane integrity of sperm in the semen of purebred Duroc and Pietrain boars should be evaluated more often during storage than in the semen of Duroc × Pietrain crossbred boars. This study provides valuable information for the development and implementation of semen quality monitoring in crossbred boars and boars of parent breeds during storage at 17 °C with respect to the cell membrane structure of sperm heads.

## Figures and Tables

**Table 1:** Frequency of the integrity of the sperm membrane as determined by the SYBR-14/PI staining method in the semen of Duroc × Pietrain crossbred boars and purebred Duroc and Pietrain boars depending on the storage time and heterosis effects (mean ± SEM).

Semen Storage Time (Hour)	Breed			Heterosis Effects VR (%)
	Duroc × Pietrain	Duroc	Pietrain	
1	89.19 ^a^ ± 0.82	83.12 ^b^ ± 2.12	84.69 ^b^ ± 1.02	6.29
		X_MP_ = 83.91		
24	86.87 ^a^ ± 1.43	80.75 ^b^ ± 2.41	83.06 ^a,b^ ± 1.83	9.05
		X_MP_ = 81.91		
48	80.37 ^a^ ± 1.45	69.05 ^b^ ± 3.20	79.25 ^c^ ± 2.15	8.39
		X_MP_ = 74.15		
72	78.87 ^a^ ± 1.58	67.18 ^b^ ± 3.16	75.06 ^c^ ± 2.34	10.90
		X_MP_ = 71.12		
96	74.06 ^a^ ± 1.51	62.31 ^b^ ± 3.06	70.56 ^c^ ± 1.94	11.47
		X_MP_ = 66.44		

^a,b,c^ Different letters in rows indicate differences (*p* ≤ 0.05).

**Table 2:** Frequency of the integrity of the sperm membrane as determined by the eosin–nigrosin staining method in the semen of Duroc × Pietrain crossbred boars and purebred Duroc and Pietrain boars depending on the storage time and heterosis effects (mean ± SEM).

Semen Storage Time (Hour)	Breed			Heterosis Effects VR (%)
	Duroc × Pietrain	Duroc	Pietrain	
1	92.75 ^a^ ± 0.36	84.68 ^b^ ± 3.89	86.87 ^b^ ± 1.20	8.13
		X_MP_ = 85.78		
24	91.02 ^a^ ± 0.65	82.68 ^b^ ± 4.22	83.43 ^b^ ± 1.78	9.60
		X_MP_ = 83.95		
48	90.37 ^a^ ± 0.75	80.50 ^b^ ± 4.75	80.06 ^b^ ± 1.15	12.57
		X_MP_ = 80.28		
72	88.37 ^a^ ± 0.51	75.62 ^b^ ± 4.65	77.87 ^b^ ± 0.81	15.14
		X_MP_ = 76.75		
96	82.25 ^a^ ± 0.83	71.56 ^b^ ± 4.82	74.44 ^c^ ± 0.99	12.67
		X_MP_ = 73.00		

^a,b,c^ Different letters in rows indicate differences (*p* ≤ 0.05).

**Table 3:** Frequency of sperm with an abnormal cell membrane structure (dead) as determined by the SYBR-14/PI staining method in the semen of Duroc × Pietrain crossbred boars and purebred Duroc and Pietrain boars depending on the storage time and heterosis effects (mean ± SEM).

Semen Storage Time (Hour)	Breed			Heterosis Effects VR (%)
	Duroc × Pietrain	Duroc	Pietrain	
1	8.68 ^a^ ± 0.61	10.56 ^a^ ± 4.36	7.62 ^a^ ± 1.31	−4.51
		X_MP_ = 9.09		
24	10.43 ^a^ ± 1.12	12.13 ^a^ ± 5.03	7.56 ^b^ ± 1.83	5.99
		X_MP_ = 9.84		
48	15.69 ^a^ ± 1.22	20.88 ^b^ ± 6.42	10.68 ^b^ ± 2.60	−2.22
		X_MP_ = 15.78		
72	16.44 ^a^ ± 1.40	24.94 ^b^ ± 5.63	13.00 ^b^ ± 2.76	−13.34
		X_MP_ = 18.97		
96	20.31 ^a^ ± 1.23	29.25 ^b^ ± 5.63	16.88 ^a^ ± 2.35	−11.96
		X_MP_ = 23.07		

^a,b^ Different letters in rows indicate differences (*p* ≤ 0.05).

**Table 4:** Frequency of moribund sperm as determined by the SYBR-14/PI staining method in the semen of Duroc × Pietrain crossbred boars and purebred Duroc and Pietrain boars depending on the storage time and heterosis effects (mean ± SEM).

Semen Storage Time (Hour)	Breed			Heterosis Effects VR (%)
	Duroc × Pietrain	Duroc	Pietrain	
1	2.12 ^a^ ± 0.44	6.31 ^b^ ± 1.55	7.62 ^b^ ± 1.52	−69.54
		X_MP_ = 6.96		
24	2.81 ^a^ ± 0.49	7.13 ^b^ ± 2.23	9.37 ^b^ ± 2.09	−65.94
		X_MP_ = 8.25		
48	3.94 ^a^ ± 0.45	9.63 ^b^ ± 2.72	10.06 ^b^ ± 2.12	−60.00
		X_MP_ = 9.85		
72	4.69 ^a^ ± 0.39	7.88 ^b^ ± 2.04	11.94 ^c^ ± 1.83	−52.67
		X_MP_ = 9.91		
96	5.63 ^a^ ± 0.64	8.43 ^b^ ± 1.66	12.56 ^c^ ± 1.80	−46.33
		X_MP_ = 10.49		

^a,b,c^ Different letters in rows indicate differences (*p* ≤ 0.05).

## Data Availability

The data presented in this study are available on request from the corresponding author.
